# Supplementary material for: The Digital Brain Bank, an open access platform for post-mortem imaging datasets
Source: eLife. 2022 Mar 17;11:e73153. doi: 10.7554/eLife.73153 (PMC9042233; doi:10.7554/eLife.73153)
Supplement: Transparent reporting form [file elife-73153-transrepform1.docx]

***eLife’s* transparent reporting form**

We encourage authors to provide detailed information *within their submission* to facilitate the interpretation and replication of experiments. Authors can upload supporting documentation to indicate the use of appropriate reporting guidelines for health-related research (see [EQUATOR Network](http://www.equator-network.org/%20)), life science research (see the [BioSharing Information Resource](https://biosharing.org/)), or the [ARRIVE guidelines](http://www.plosbiology.org/article/info:doi/10.1371/journal.pbio.1000412) for reporting work involving animal research. Where applicable, authors should refer to any relevant reporting standards documents in this form.

If you have any questions, please consult our Journal Policies and/or contact us: [editorial@elifesciences.org](mailto:editorial@elifesciences.org).

**Sample-size estimation**

- You should state whether an appropriate sample size was computed when the study was being designed
- You should state the statistical method of sample size computation and any required assumptions
- If no explicit power analysis was used, you should describe how you decided what sample (replicate) size (number) to use

Please outline where this information can be found within the submission (e.g., sections or figure legends), or explain why this information doesn’t apply to your submission:

This manuscript describes a new resource for hosting post-mortem datasets associated with existing or ongoing studies, the *Digital Brain Bank*. The number of samples provided with each dataset is provided in Table 1 in the manuscript. As the manuscript describes a data resource rather than a specific study, further details of sample size estimation outlined above do not apply to our submission.

**Replicates**

- You should report how often each experiment was performed
- You should include a definition of biological versus technical replication
- The data obtained should be provided and sufficient information should be provided to indicate the number of independent biological and/or technical replicates
- If you encountered any outliers, you should describe how these were handled
- Criteria for exclusion/inclusion of data should be clearly stated
- High-throughput sequence data should be uploaded before submission, with a private link for reviewers provided (these are available from both GEO and ArrayExpress)

Please outline where this information can be found within the submission (e.g., sections or figure legends), or explain why this information doesn’t apply to your submission:

Details of exclusion/inclusion criteria for datasets uploaded to the *Digital Brain Bank* resource are provided in the *Methods* section under the *Datasets*, *Conditions for Data Uploading* and *Types of Data Provided* subheadings. As the manuscript describes a data resource rather than a specific study, further details of replicates outlined above do not apply to our submission.

**Statistical reporting**

- Statistical analysis methods should be described and justified
- Raw data should be presented in figures whenever informative to do so (typically when N per group is less than 10)
- For each experiment, you should identify the statistical tests used, exact values of N, definitions of center, methods of multiple test correction, and dispersion and precision measures (e.g., mean, median, SD, SEM, confidence intervals; and, for the major substantive results, a measure of effect size (e.g., Pearson's r, Cohen's d)
- Report exact p-values wherever possible alongside the summary statistics and 95% confidence intervals. These should be reported for all key questions and not only when the p-value is less than 0.05.

Please outline where this information can be found within the submission (e.g., sections or figure legends), or explain why this information doesn’t apply to your submission:

As the manuscript describes a data resource rather than a specific study, details of statistical analysis outlined above do not apply to our submission. However, to demonstrate the potential of the data provided in the *Digital Brain Bank* resource, a single example cohort analysis was performed. Results of this analysis are provided in *Figure 3c*. Details of the methodology and statistical analysis for this cohort analysis are provided in the *Supporting Information* under the *Human ALS MRI-Histology Callosum analysis* subheading.

(For large datasets, or papers with a very large number of statistical tests, you may upload a single table file with tests, Ns, etc., with reference to sections in the manuscript.)

**Group allocation**

- Indicate how samples were allocated into experimental groups (in the case of clinical studies, please specify allocation to treatment method); if randomization was used, please also state if restricted randomization was applied
- Indicate if masking was used during group allocation, data collection and/or data analysis

Please outline where this information can be found within the submission (e.g., sections or figure legends), or explain why this information doesn’t apply to your submission:

One cohort dataset provided in the first release to the *Digital Brain Bank* (*Human ALS MRI-Histology* dataset) contains two groups of post-mortem brains (control and ALS brains). These brains were allocated to control and ALS groups by the Oxford Brain Bank, as noted in the *Results* section (*Digital Pathologist* subheading).

**Additional data files (“source data”)**

- We encourage you to upload relevant additional data files, such as numerical data that are represented as a graph in a figure, or as a summary table
- Where provided, these should be in the most useful format, and they can be uploaded as “Source data” files linked to a main figure or table
- Include model definition files including the full list of parameters used
- Include code used for data analysis (e.g., R, MatLab)
- Avoid stating that data files are “available upon request”

Please indicate the figures or tables for which source data files have been provided:

The *Digital Brain Bank* is a data resource. All datasets described in this manuscript (*Table 1*) are available through the *Digital Brain Bank*, with details of access provided within the manuscript (*Results* section - *Requirements for Data Access and Referencing Datasets* subheading) and on the website ([https://open.win.ox.ac.uk/DigitalBrainBank/](https://open.win.ox.ac.uk/DigitalBrainBank/#/)).

Code for the *Digital Brain Bank* resource is available through GitLab (<https://git.fmrib.ox.ac.uk/thanayik/dbb>), linked in the manuscript (*Results* section – *Tview* subheading) and on the *Digital Brain Bank* website.

When available, details of associated processing code for each dataset (*Table 1*) is linked to the dataset's Information page on the *Digital Brain Bank* website, as noted in the manuscript (*Methods* section - *Types of Data Provided* subheading) .

Source data for the corpus callosum analysis in Fig 3c is additionally provided as a Supplementary File (*Figure 3c – Source Data*).
